# Choroidal Thickness and Ganglion Cell Complex in Pubescent Children with Type 1 Diabetes without Diabetic Retinopathy Analyzed by Spectral Domain Optical Coherence Tomography

**DOI:** 10.1155/2018/5458015

**Published:** 2018-04-03

**Authors:** Joanna Gołębiewska, Andrzej Olechowski, Marta Wysocka-Mincewicz, Marta Baszyńska-Wilk, Artur Groszek, Agnieszka Czeszyk-Piotrowicz, Mieczysław Szalecki, Wojciech Hautz

**Affiliations:** ^1^Department of Ophthalmology, The Children's Memorial Health Institute, Aleja Dzieci Polskich 20, Warsaw, Poland; ^2^Department of Diabetology and Endocrinology, The Children's Memorial Health Institute, Aleja Dzieci Polskich 20, Warsaw, Poland; ^3^Department of Medicine and Health Sciences, UJK, Kielce, Poland

## Abstract

**Aim:**

To assess the retinal and choroidal thickness and ganglion cell complex (GCC) in pubescent children with type 1 diabetes (T1D) without diabetic retinopathy (DR), using spectral domain optical coherence tomography (SD-OCT).

**Materials and Method:**

Sixty-four right eyes of 64 subjects with T1D and 45 right eyes of 45 age-matched healthy volunteers (control group) were enrolled in this study. The mean age of the subjects and controls was 15.3 (±SD = 2.2) and 14.6 (±SD = 1.5), respectively. SD-OCT was performed using RTVue XR Avanti. Ganglion cell complex (GCC), GCC focal loss volume (FLV), GCC global loss volume (GLV), choroidal thickness (CT), foveal (FT) and parafoveal thickness (PFT), and foveal (FV) and parafoveal volume (PFV) data were analyzed.

**Results:**

There was no significant difference between subjects and controls in the CT in the fovea and nasal, temporal, superior, and inferior quadrants of the macula. There were no significant correlations between CT, duration of diabetes, and HbA1C level (*p* = 0.272 and *p* = 0.197, resp.). GCC thickness did not differ significantly between the groups (*p* = 0.448), but there was a significant difference in FLV (*p* = 0.037). Significant differences between the groups were found in the PFT and PFV (*p* = 0.004 and *p* = 0.005, resp.). There was a significant negative correlation between PFT, PFV, and HbA1C level (*p* = 0.002 and *p* = 0.001, resp.).

**Conclusions:**

Choroidal thickness remains unchanged in children with T1D. Increased GCC FLV might suggest an early alteration in neuroretinal tissue. Parafoveal retinal thickness is decreased in pubescent T1D children and correlates with HbA1C level. OCT can be considered a part of noninvasive screening in children with T1D and a tool for early detection of retinal and choroidal abnormalities. Further OCT follow-up is needed to determine whether any of the discussed OCT measurements are predictive of future DR severity.

## 1. Introduction

Diabetes mellitus (DM) is the third most common chronic disease in children. The majority of cases are type 1 diabetes (T1D), but the global obesity epidemic contributes to the increasing incidence of type 2 diabetes (T2D) in children and adolescents [1–3]. Most previous studies focused on diabetic retinopathy (DR) as an increasingly prevalent disease and the leading cause of blindness in working-age individuals in industrialized countries [4–6]. Far less attention has been paid to choroidal vasculopathy, despite clinical and experimental findings implicating choroidal blood flow in the pathogenesis of diabetic retinopathy [7–9]. Macular choroidal thickness (CT) is considered a putative measure of choroidal blood flow. Some researchers found decreased choroidal thickness in diabetic patients, whereas others reported increased or unchanged CT in various stages of DR [10–14]. Thus, the status of the choroid in patients with DM remains controversial.

Various studies using electroretinography, colour vision, and contrast sensitivity testing reported neural tissue loss, in particular affecting retinal ganglion cells, apoptosis of retinal glial and neural cells, and decreased thickness of inner retinal layers before DR becomes clinically detectable [15–17]. The exact mechanism for inner retinal loss is not clear and some authors investigated the relationship between diabetic peripheral neuropathy (DPN) and retinal tissue thickness [18, 19]. Therefore, early detection of choroidal vasculopathy and neuropathy in T1D patients through screening programs may be crucial for commencing treatment before the onset of DR.

Indirect ophthalmoscopy and stereoscopic dilated fundus photography, a practice commonly used worldwide, offers high diagnostic accuracy in DR detection [20]. Optical coherence tomography is a noninvasive tool enabling the reproducible and quantitative assessment of the retinal layers, which currently remains the most precise method to measure retinal and choroidal thicknesses *in vivo*. To date, there is a number of studies to discuss OCT findings in adults with diabetes mellitus and only a few reports to discuss OCT findings in DM pediatric population [21–27].

The aim of our study was to assess retinal and choroidal thickness and ganglion cell complex in pubescent T1D children.

## 2. Material and Methods

This cross-sectional study was approved by the Institutional Review Board and followed the tenets of the Declaration of Helsinki. After explanation of the nature and possible consequences of the study, a written informed consent was obtained from the patient's legal guardian and from patients above 16 years of age.

Sixty-four T1D children at the age of 11–18 years, without signs of DR and with diabetes duration of 1 year or more, were enrolled in the study. Exclusion criteria were history of prematurity and other concomitant retinal pathologies, such as hereditary retinal dystrophies, vitreoretinal diseases, as well as uveitis, glaucoma, history of peripheral neuropathy, and high refractive error (spheric equivalent > +/−3.00 diopters). Eyes with poor-quality scans, due to unstable fixation, were also excluded. Controls were defined as having a normal finding in ocular examination and no history of diabetes. They were age- and sex-matched to the patients.

Every patient underwent a complete ocular examination, including best-corrected visual acuity (BVCA), slit lamp biomicroscopy, dilated fundus examination, and colour fundus photography. Clinical data recorded for each diabetic subject included duration of diabetes, systolic and diastolic blood pressure, mean and actual levels of glycated hemoglobin (HbA1C), daily urine creatinine excretion, serum creatinine levels, as well as mean and actual levels of daily urine albumin excretion.

SD-OCT was performed in both subjects and controls using a commercially available RTVue XR Avanti (Optovue, Fremont, CA, USA). Foveal thickness (FT) (*μ*m), parafoveal thickness (PFT) (*μ*m), foveal volume (FV) (mm^3^), and parafoveal volume (PFV) data were obtained from retinal maps, using the same device. All acquired images were inspected, and if automatic segmentation errors occurred or resulted in measurement artifacts, manual segmentation was performed. All OCT scans with motion artifacts were excluded.

**Figure 1:**
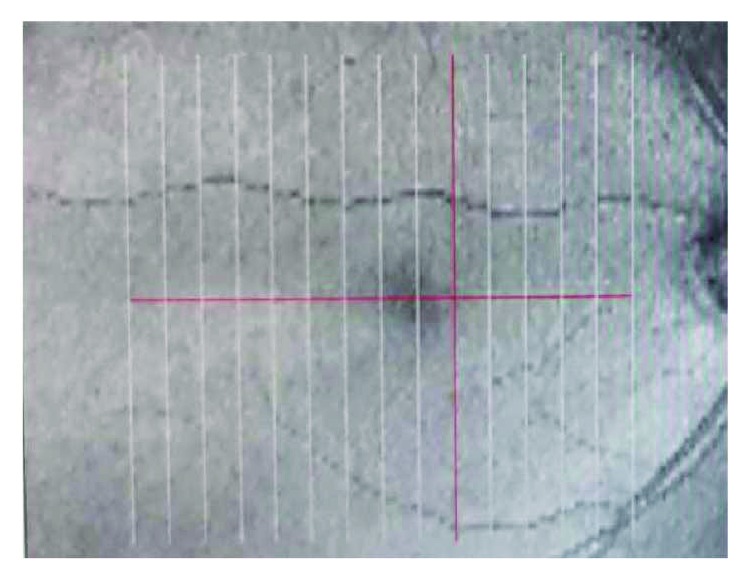
The ganglion cell complex scan pattern (7 × 7 mm) consists of 15 vertical and 1 horizontal scan lines.

**Figure 2:**
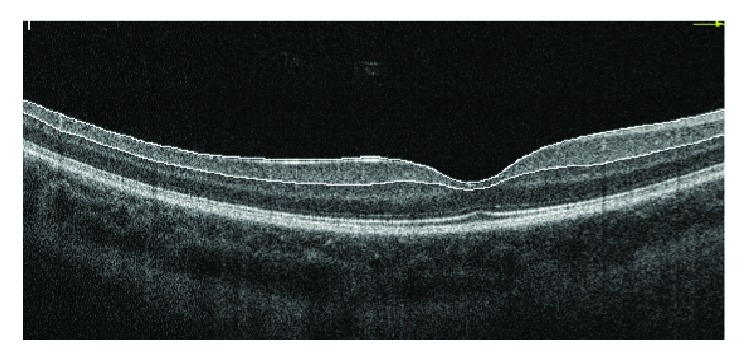
An example of horizontal macular OCT cross section in a T1D patient. The GCC thickness is automatically measured as the distance between the ILM and the outer IPL boundary.

GCC scan protocol (formerly called MM7 scan), which consists of one horizontal line with a 7 mm scan length (934 A scans) and 15 vertical lines with a 7 mm scan length and a 0.5 mm interval (800 A scans) centered at 1 mm temporally to the fovea, was used in all participants. GCC thickness, defined as the distance from the internal limiting membrane to the outer boundary of the inner plexiform layer, was calculated automatically by the device (Figures 1 and 2).

Eyes were divided into two sectors, superior and inferior. GCC was expressed as the average thickness of both sectors (Avg GCC) and separately as the thickness of the superior (Sup GCC) and inferior (Inf GCC) sector. Furthermore, the RTVue SD-OCT device is equipped with software enabling the analysis of diffuse and focal GCC defects by calculating global loss volume (GLV) and focal loss volume (FLV), respectively. These parameters, developed and introduced by Tan et al., provide a quantitative measurement of change in the GCC volume [28]. The GLV measures the average amount of GCC volume loss over the entire recorded GCC field and corresponds to the mean deviation (MD) values in visual field tests. The GLV can best detect diffuse ganglion cell loss. The FLV detects focal losses to correct for overall absolute changes and corresponds to the corrected pattern standard deviation in the visual field. GLV is calculated from the fractional deviation map showing the percentage of GCC thickness decrease at each pixel location compared with the expected or normal values. The FLV is determined based on a normalized pattern map by dividing GCC thickness values at each location by the average GCC thickness from the entire map for a given individual. All significant differences between the pattern map created for an individual and pattern map of the normative database yields the pattern deviation map.

A crossline scan was performed to obtain high-quality images of the retina and choroid. Choroidal thickness was measured manually using the built-in calipers in OCT software. Choroidal thickness, defined as the distance between the hyperreflective line corresponding to the outer boundary of the RPE and the hyperreflective line corresponding to the chorioscleral interface, was measured three times by two independent researchers. Mean values were recorded and included in the analysis. The measurements were obtained in the subfoveal region and at a distance of 1500 *μ*m superiorly, inferiorly, nasally, and temporally from this site. All measurements were performed at the same time of the day (between 9:00 am and 11:00 am) in all children to avoid the effect of diurnal CT variation on the results. Only measurements from right eyes of both subjects and controls were included in the analysis.

## 3. Statistical Analysis

The variables were expressed as means, standard deviations, 95% confidence intervals, and ranges. The one-way multifactor analysis of variance (ANOVA) was used to determine the differences between patients and controls, if the assumptions of normality of distribution and homogeneity of variances were met, or generalized linear models with robust standard errors, when said assumptions were violated. Linear relationships between selected quantitative variables were assessed using the Pearson product-moment correlation coefficient. Glass's Δ traditionally estimated for experimental case-control studies using a control group standard deviation rather than a pooled standard deviation was used as a measure of effect size [29]. A standard deviation from the nondiabetic group was chosen due to its lower variability and better stability. A level of *p* < 0.05 was considered statistically significant for all comparisons. All statistical computations were carried out using Stata/Special Edition, release 14.2 (StataCorp LP, College Station, Texas, USA).

## 4. Results

Sixty-four right eyes of 64 subjects with T1D and 45 right eyes of 45 age-matched healthy volunteers (controls) were enrolled in this study. The mean age in the study group was 15.3 (±SD = 2.2) years and in the control group 14.6 (±SD = 1.5) years. All subjects had 20/20 vision and normal colour fundus photographs. The in-depth descriptive characteristics of the entire cohort are shown in Table 1.

Table 2 shows descriptive measures of the choroidal thickness (*μ*m) in the studied patients by presence of T1D. CT in the fovea and nasal, temporal, superior, and inferior quadrants of the macula did not differ statistically between the study groups: *p* = 0.134, *p* = 0.270, *p* = 0.691, *p* = 0.504, and *p* = 0.862, respectively.

**Figure 3:**
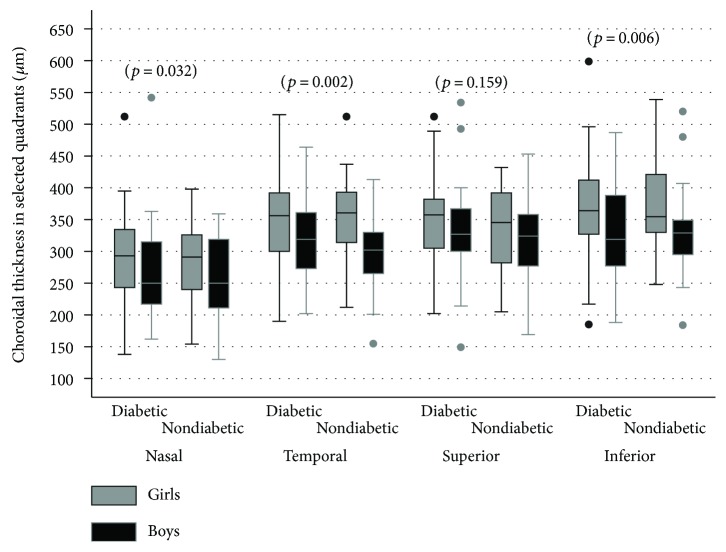
Central tendency and dispersion for the choroidal thickness (*μ*m) in selected retinal quadrants in the study sample by T1D status and gender.

However, regardless of the prevalence of T1D in the studied children, the CT was significantly thicker in girls than in boys, except for the superior quadrant Figure 3.

There were no significant correlations between CT, duration of diabetes, and HbA1C level (*r* = −0.16, *p* = 0.272 and *r* = −0.22, *p* = 0.197, resp.).

GCC thickness did not differ significantly between the groups (*p* = 0.448), but there was a significant difference between FLV (*p* = 0.037) (Table 3).

Effect size measures

- FLV, Glass's Δ = −0.548 (95% CI: −0.953 to −0.136).

There was no significant correlation between GCC and duration of diabetes (*r* = −0.05, *p* = 0.696). There were no significant differences in foveal thickness and foveal volume between the study groups (*p* = 0.206 and *p* = 0.197, resp.).

Statistically significant differences were found in the PFT and PFV between the groups (*p* = 0.004 and *p* = 0.005, resp.) (Table 4).

Effect size measures

- parafoveal thickness, Glass's Δ = 0.609 (95% CI: 0.194–1.018),
- parafoveal volume, Glass's Δ = 0.601 (95% CI: 0.186–1.009).

**Figure 4:**
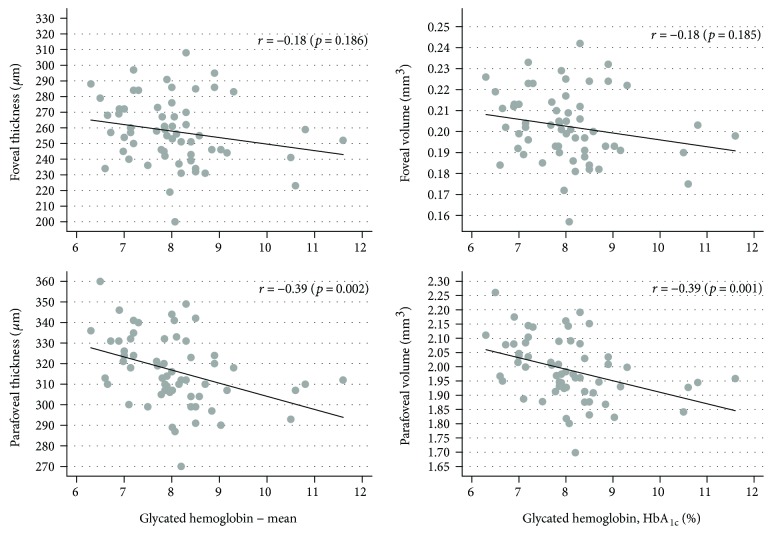
Correlations between the level of glycated hemoglobin (HbA_1c_, %) and foveal/parafoveal thickness (*μ*m) and volume (mm^3^) in the studied patients with type 1 diabetes mellitus.

There was a significant correlation between PFT, PFV, and HbA1C level (Figure 4).

## 5. Discussion

Several studies over the past 20–30 years have shown a declining incidence of DR in children with diabetes, from 49% in the early 1990s to about 12–20% in the 2000s. This may be the result of more effective treatment, the use of the insulin pumps, and better education of the affected children and their families [30, 31]. In our study group, no child had any signs of diabetic retinopathy on fundus examination and colour fundus photography. The risk of developing retinopathy in youths is low before puberty. Adolescents with type 1 diabetes are faced with the significant challenge of regulating their blood sugar levels. Olsen et al. noted that the risk of DR increases twice in postpubertal years as compared to prepubertal years, which may be due to increasing hemoglobin A1c levels during puberty [32]. Since a group of pubescent children has a higher risk of developing DR, we decided to choose them for our research.

To the best of our knowledge, this is the first study to evaluate OCT data, which focused only on pubescent T1D children. Histological studies show loss of choriocapillaries in patients with DM, which results in reduced choroidal blood flow, retinal tissue hypoxia, as well as retinal pigment epithelium and photoreceptor dysfunction and death [10]. Regatieri et al. showed a significant decrease in CT in patients with diabetic macular oedema and those treated for proliferative diabetic retinopathy as compared to healthy volunteers. They concluded that reduced CT may have been related to the severity of retinopathy [21]. Sheth et al. found a significant reduction in CT in the eyes with ischemic diabetic maculopathy as compared to nonischemic DR and diabetic population without DR, which may explain the visual loss in ischemic maculopathy [33]. Our results do not support these findings, as we did not find significant differences in choroidal thickness between the groups. It should be noted, though, that whereas the former studies were carried out in adults, ours was carried out in children. Sayin et al. proved that CT of diabetic children is similar to healthy controls and is not affected by HbA1c, age, or duration of diabetes [27]. Their results, consistent with ours, might suggest unaffected choroidal blow flow in T1D children, without DR. Furthermore, in our study, girls had significantly thicker choroid than boys, regardless of T1D prevalence. The significance of this finding remains uncertain at the moment. Other authors suggested a biological explanation of this phenomenon, linked to a different hormonal exposure between males and females. The previous studies proved that sex and hormonal status influence the choroidal blood flow, probably due to the vascular effect of oestrogens and progestins [34, 35]. Further follow-up of these children is necessary in order to assess the clinical significance of these findings and to determine their potential effect on the DR onset. The exact relationship between DR and diabetic retinal neuropathy has not been fully explained yet. Some researchers have found that retinal neurodegeneration may occur in DM before any microcirculatory abnormalities become detectable [24]. They are also likely to occur in an independent manner [36–40]. The mechanism for inner retinal loss in DM patients may be explained by lower perfusion and higher metabolic demands of the inner retina, which is vulnerable to disease-induced metabolic stress [41]. Objective assessment of GCC finds importance in detection of inner retinal loss in patients with DM. Several studies reported GCC thinning in patients with DM without DR [23, 24]. Our results are consistent with Srinivasan, who found an abnormality in GCC FLV in affected subjects and confirmed that the loss of neural tissue begins in the early stages of diabetes as an independent predictor of diabetic peripheral neuropathy [39]. El-Fayoumi reported significant reduction in the average GCC thickness at the macula in 46 children with T1D with normal fundus examination. Similarly, in our study, there was no correlation between GGC thickness and HbA1c, duration of DM, and the age of DM onset [26]. Pierro et al. found decreased GCC and choroidal thickness only in patients with T2D, not T1D and suggested that insulin resistance might be one of the causes of neurodegeneration [41]. Most reports reveal that the retina becomes thinner in DR [13, 22, 40]. We confirm these results, as there were significant differences in PFT and PFV between the groups. However, unlike most previous studies, which show that macular thickness decreases due to neural tissue loss, the decreased PFT and PFV are not related to the decreased GCC thickness in our group of T1D patients. We agree with Srinivasan et al. who hypothesise that microglial changes, which can occur prior to neuronal cell death may be the reason of decreased PFT and thinner parafovea may proceed detectable changes in neuroretinal layer thickness [40]. The short duration of diabetes in our adolescent patients is a likely explanation for the absence of significant GCC thinning. The measurements of PFT and PFV, albeit of secondary importance to our study, appear interesting and require further research in a larger number of eyes. In the present study, pubescent youths with T1D presented with significantly fewer objective signs of retinal and choroidal structure impairment than diabetic adults as previously reported [10–13, 36–38, 41]. We are hoping to continue the follow-up of these young patients to monitor the status of their retinas and choroids.

The limitation of the current study is poor sample representativeness. It is a single-center study with monoracial background, as all subjects were Caucasian, and the uniformity of this clinical population may not exactly reflect the entire cohort of diabetic children worldwide.

## 6. Conclusions

Choroidal thickness remains unchanged in children with T1D. Furthermore, it is significantly thicker in girls than in boys, regardless of the prevalence of T1D. Increased GCC FLV might suggest an early alteration in neuroretinal tissue in these patients. Parafoveal retinal thickness is decreased in pubescent T1D children and correlates with HbA1C level. Based on these findings, OCT can be considered a part of noninvasive screening in children with T1D and a tool for early detection of retinal and choroidal abnormalities. Further OCT follow-up is needed to determine whether any of the discussed OCT measurements are predictive of future DR severity.

## Figures and Tables

**Table 1:** Study sample characteristics.

Variable	*M*	SD	95% CI	Min.–max.
Age (years)				
Diabetic patients Nondiabetic patients	15.3 14.6	2.2 1.6	14.7–15.8 14.1–15.0	11.3–18.5 13.0–18.0
Diabetes duration (years)	6.3	3.4	5.5–7.2	1.0–14.4
Age at onset (years)	8.9	3.8	8.0–9.0	2.3–16.5
Glycated hemoglobin − actual level (%)	8.2	1.2	7.9–8.5	6.4–13.3
Glycated hemoglobin − mean level (%)	8.1	1.1	7.8–8.3	6.3–11.6
Actual level of daily urine albumin excretion (mg/d)	10.3	6.9	8.5–12.2	0.5–33.3
Mean level of daily urine albumin excretion (mg/d)	8.9	8.1	6.7–11.1	0.5–40.5
Serum creatinine (mg/dL)	0.69	0.16	0.65–0.73	0.42–1.12
Daily urine creatinine excretion (mg/d)	1.29	0.95	1.01–1.58	0.27–5.47
Systolic blood pressure (mmHg)	114	10.8	111.0–116.9	84–140
Diastolic blood pressure (mmHg)	68	10.5	65.3–71.1	40–91

M: mean; SD: standard deviation; CI: confidence interval.

**Table 2:** Descriptive statistics for choroidal thickness (CT, *μ*m) in individual retinal quadrants (diabetic patients versus nondiabetic).

Variable	Study group	*M*	SD	95% CI	Range	*p*
Central choroidal thickness, CT (*μ*m)	Diabetic	355.65	77.50	335.09–376.21	187–585	0.081
	Nondiabetic	327.98	66.02	307.66–248.29	179–479	
CT—nasal quadrant (*μ*m)	Diabetic	282.32	76.59	261.99–302.64	138–542	0.427
	Nondiabetic	267.16	68.68	246.03–288.30	130–398	
CT—temporal quadrant (*μ*m)	Diabetic	338.88	71.18	319.99–357.76	190–515	0.338
	Nondiabetic	324.07	70.17	302.47–345.66	155–512	
CT—superior quadrant (*μ*m)	Diabetic	342.40	76.48	321.72–363.08	149–534	0.418
	Nondiabetic	328.70	70.11	307.12–350.27	169–453	
CT—inferior quadrant (*μ*m)	Diabetic	352.35	82.17	330.13–374.56	185–599	0.813
	Nondiabetic	354.56	78.15	330.51–378.61	184–539	

M: mean; SD: standard deviation; CI: confidence interval.

**Table 3:** Descriptive statistics of GCC parameters in the studied patients by presence of type 1 diabetes mellitus.

Variable	Study group	*M*	SD	95% CI	Range	*p*
Ganglion cell complex—total	Diabetic	97.51	6.77	95.77–99.24	80.97–118.39	0.448
	Nondiabetic	98.08	6.03	96.25–99.92	87.23–111.94	
GCC—superior quadrant	Diabetic	96.05	10.45	93.38–98.73	30.88–117.00	0.371
	Nondiabetic	97.42	6.56	95.42–99.41	86.46–111.43	
GCC—inferior quadrant	Diabetic	98.05	8.49	95.88–100.23	81.69–138.14	0.401
	Nondiabetic	98.74	5.74	96.99-100.48	88.00–112.47	
Focal loss volume (%)	Diabetic	0.512	0.756	0.313–0.711	0.000–4.420	**0.037**
	Nondiabetic	0.275	0.434	0.142–0.407	0.000–2.202	
Global loss volume (%)	Diabetic	2.349	2.926	1.580–3.118	0.000–14.402	0.282
	Nondiabetic	1.842	1.909	1.262–2.422	0.003–8.265	

M: mean; SD: standard deviation; CI: confidence interval.

**Table 4:** Descriptive statistics foveal/parafoveal thickness (*μ*m) and volume (mm^3^) in the studied patients by presence of type 1 diabetes mellitus.

Variable	Study group	*M*	SD	95% CI	Range	*p*
Foveal thickness (*μ*m)	Diabetic	257.82	21.37	252.44–263.21	200–308	*p* = 0.206
	Nondiabetic	262.63	20.07	256.45–268.80	221–324	
Parafoveal thickness (*μ*m)	Diabetic	315.90	17.95	311.38–320.42	270–360	**p** = 0.004
	Nondiabetic	325.26	15.37	320.47–330.05	289–360	
Foveal volume (mm^3^)	Diabetic	0.202	0.017	0.198–0.207	0.157–0.242	*p* = 0.197
	Nondiabetic	0.206	0.016	0.201–0.211	0.173–0.254	
Parafoveal volume (mm^3^)	Diabetic	1.985	0.112	1.957–2.014	1.698–2.261	**p** = 0.005
	Nondiabetic	2.043	0.096	2.013–2.073	1.818–2.261	

^∗^Patient age and gender were considered in the multivariate analyses.
